# Proteasome Inhibitors Activate Autophagy Involving Inhibition of PI3K-Akt-mTOR Pathway as an Anti-Oxidation Defense in Human RPE Cells

**DOI:** 10.1371/journal.pone.0103364

**Published:** 2014-07-25

**Authors:** Bingrong Tang, Jingjing Cai, Lin Sun, Yiping Li, Jia Qu, Barbara Joy Snider, Shengzhou Wu

**Affiliations:** 1 School of Optometry and Ophthalmology and Eye Hospital, Wenzhou Medical University, Wenzhou, Zhejiang Province, P.R. China; 2 State Key Laboratory Cultivation Base and Key Laboratory of Vision Science, Ministry of Health and Zhejiang Provincial Key Laboratory of Ophthalmology and Optometry, Wenzhou, Zhejiang Province, P.R. China; 3 Laboratory of Molecular Cell Biology, Institute of Biochemistry and Cell Biology, Shanghai Institutes for Biological Sciences, Chinese Academy of Sciences, Shanghai, P.R. China; 4 Department of Neurology, Washington University School of Medicine, St. Louis, Missouri, United States of America; University of Cambridge, United Kingdom

## Abstract

The two major intracellular protein degradation systems, the ubiquitin-proteasome system (UPS) and autophagy, work collaboratively in many biological processes including development, apoptosis, aging, and countering oxidative injuries. We report here that, in human retinal pigment epithelial cells (RPE), ARPE-19 cells, proteasome inhibitors, clasto-lactacystinβ-lactone (LA) or epoxomicin (Epo), at non-lethal doses, increased the protein levels of autophagy-specific genes Atg5 and Atg7 and enhanced the conversion of microtubule-associated protein light chain (LC3) from LC3-I to its lipidative form, LC3-II, which was enhanced by co-addition of the saturated concentration of Bafilomycin A1 (Baf). Detection of co-localization for LC3 staining and labeled-lysosome further confirmed autophagic flux induced by LA or Epo. LA or Epo reduced the phosphorylation of the protein kinase B (Akt), a downstream target of phosphatidylinositol-3-kinases (PI3K), and mammalian target of rapamycin (mTOR) in ARPE-19 cells; by contrast, the induced changes of autophagy substrate, p62, showed biphasic pattern. The autophagy inhibitor, Baf, attenuated the reduction in oxidative injury conferred by treatment with low doses of LA and Epo in ARPE-19 cells exposed to menadione (VK3) or 4-hydroxynonenal (4-HNE). Knockdown of Atg7 with siRNA in ARPE-19 cells reduced the protective effects of LA or Epo against VK3. Overall, our results suggest that treatment with low levels of proteasome inhibitors confers resistance to oxidative injury by a pathway involving inhibition of the PI3K-Akt-mTOR pathway and activation of autophagy.

## Introduction

Autophagy allows cells to adapt to nutrient deficiency and cellular injuries. It includes three main mechanisms: macroautophagy, microautophagy, and chaperone-mediated autophagy [1]. Macroautophagy (hereafter referred to as autophagy) begins with formation of autophagosome, which sequesters unused proteins and damaged cellular organelles. The autophagosome fuses with lysosome to form autolysosomes in which degradation occurs [1]. Autophagy is an orchestrated cascade that involves more than 30 autophagy-specific proteins (Atgs), conserved from yeast to mammals. For instance, autophagosome expansion, an early step in autophagy, involves insertion of LC3-II into vacuole membrane. This requires Atg7 (E1-like ubiquitin-activating enzyme), Atg3 (E2-like ubiquitin-conjugation enzymes), Atg5-Atg12-Atg16 complex (E3-like ubiquitin-ligase), and other Atgs to work in concert to conjugate phosphatidylethanolamine to LC3-I, thus forming LC3-II [2], [3]. The delicate process of starvation-induced autophagy [4] is inversely regulated by mTOR which is activated by PI3K-Akt induced by insulin or other growth factor [5], [6].

Ubiquitin-proteasome system (UPS) mediated protein degradation differs from autophagy mediated degradation in that the UPS is independent of lysosome and targets short-lived proteins while autophagy is lysosome-dependent and targets long-lived proteins or organelles. Emerging evidence suggest that there is cross-talk between these two major intracellular degradation systems; for instance, inhibition of the proteasome pathway can enhance autophagy [7], [8], [9] and inhibition of autophagy either enhances proteasome activity [10] or impairs the clearance of proteasome substrates by delaying delivery of ubiquitinated protein to proteasome [11]. Activation of the autophagy pathway can be pro-apoptotic or anti-apoptotic [12], [13], [14]; under some contexts, activation of autophagy can serve as an important defense against oxidative injuries [15], [16], [17]. We have previously reported that treatment with proteasome inhibitors can reduce oxidative injury in human RPE cells [18]. We therefore tested whether the irreversible proteasome inhibitors, LA and Epo, can activate autophagy in these cells and explored possible mechanisms for the activation of autophagy and the reduction in oxidative injury.

## Materials and Methods

### Materials

The following substances, materials, and reagents (and suppliers) were used in this study: menadione, 4',6-diamidino-2-phenylindole (DAPI), polyethyleneimine, Triton-X100 (Sigma, St. Louis, MO); clasto-lactacystin-β-lactone, 4-ydroxynonenal, and protease inhibitor cocktail (Calbiochem,San Diego, CA); cell proliferation assay (MTS, CellTiter 96 AQueous One Solution), caspase-3 activity assay kit (Promega); transfection reagents (Lipofectamine 2000; Invitrogen Life Technologies, Carlsbad, CA); clear-blue x-ray films (CL-XPosure films; Thermo Scientific Branch); antibodies, ATG5, ATG7, HDAC6, phospho-AKT, AKT, phospho-mTOR, mTOR,LC3, p62 (Cell Signaling Technology); acrylamide–bis-acrylamide solution (29∶1; Bio-Rad); and ARPE-19 cells (American Type Culture Collection [ATCC], Manassas, VA); lyso tracker, lipofectamine 2000 (Invitrogen); FITC-conjugated goat anti-mouse IgG (Beyotime, Beijing); non-specific siRNA, and ATG7 siRNA (GenePharma, Shanghai).

### Methods

#### Cell Culture

ARPE-19 cells were cultured as previously described [18].

#### Western blot analysis

ARPE-19 cells were washed once with PBS and lysed by addition of Super RIPA buffer containing a protease-inhibitor cocktail (Sigma, St Louis). The first antibodies: ATG5 (1∶1000), ATG7 (1∶1000), mTOR (1∶1000), Phospho-mTOR (1∶1000), AKT(1∶1000), Phospho-AKT (1∶1000), LC3 (1∶1000), p62 (1∶1000), and the peroxidase-conjugated secondary antibody (1∶5000) were used. Details of the protein blotting procedures were very similar to the protocol used previously [19].

#### Immunofluorescent confocal laser microscope

ARPE-19 cells were cultured on polyethyleneimine - coated coverslips sit in 6-well plates. After treated with LA, Epo, or sham-treatment, the cells were firstly labeled by incubating with lysotracker (Invitrogen), a lysosome reporter dye, for 90 min at 37°C. After washed with PBS, the cells were fixed in 4% paraformaldehyde for 5–10 min, washed in PBS, blocked in goat sera for 45 min, and then incubated with LC3 antibody (1∶250) in 0.1% Triton-X100 for 2 h following incubated with FITC-conjugated goat anti-mouse IgG in 0.1% Triton-X100 for another 45 min. Finally, the nuclei were stained with DAPI for 3 min, washed, and then observed under a Zeiss LSM 710 confocal microscope system (Carl Zeiss, Germany). The images were taken under oil-immersion lens (X 63) and processed with Zen Le software. All the procedures were performed under ambient temperature.

#### MTS Assay

MTS assay was done as previously described [18].

#### Analysis of Proteasome Activity In Vitro

Measurement of proteasome activity was performed as previously described [18]. Chymotrypsin-like degradative activity, mostly specific for enzymatic activities of proteasome complex, was used to indicate proteasome activity in the study.

#### Assessment of Caspase-3 Activity

Assay of caspase-3 activity in ARPE-19 cells followed previous procedure [20].

#### RNA Interference

ARPE-19 cells were transfected with either non-specific siRNA, or ATG7 siRNA (60 nM) under the help of lipofectamine 2000. ARPE-19 cells were then subjected to treatments, followed by western blot or MTS assay.

#### Statistical Analysis

Data were analyzed for significant difference (P<0.05) by ANOVA and Bonferroni post hoc test for multiple comparisons (SPSS 15.0.1; SPSS, Inc., Chicago, IL).

## Results

### 1. LA or Epo activated autophagy pathway in RPE

**Figure 1:**
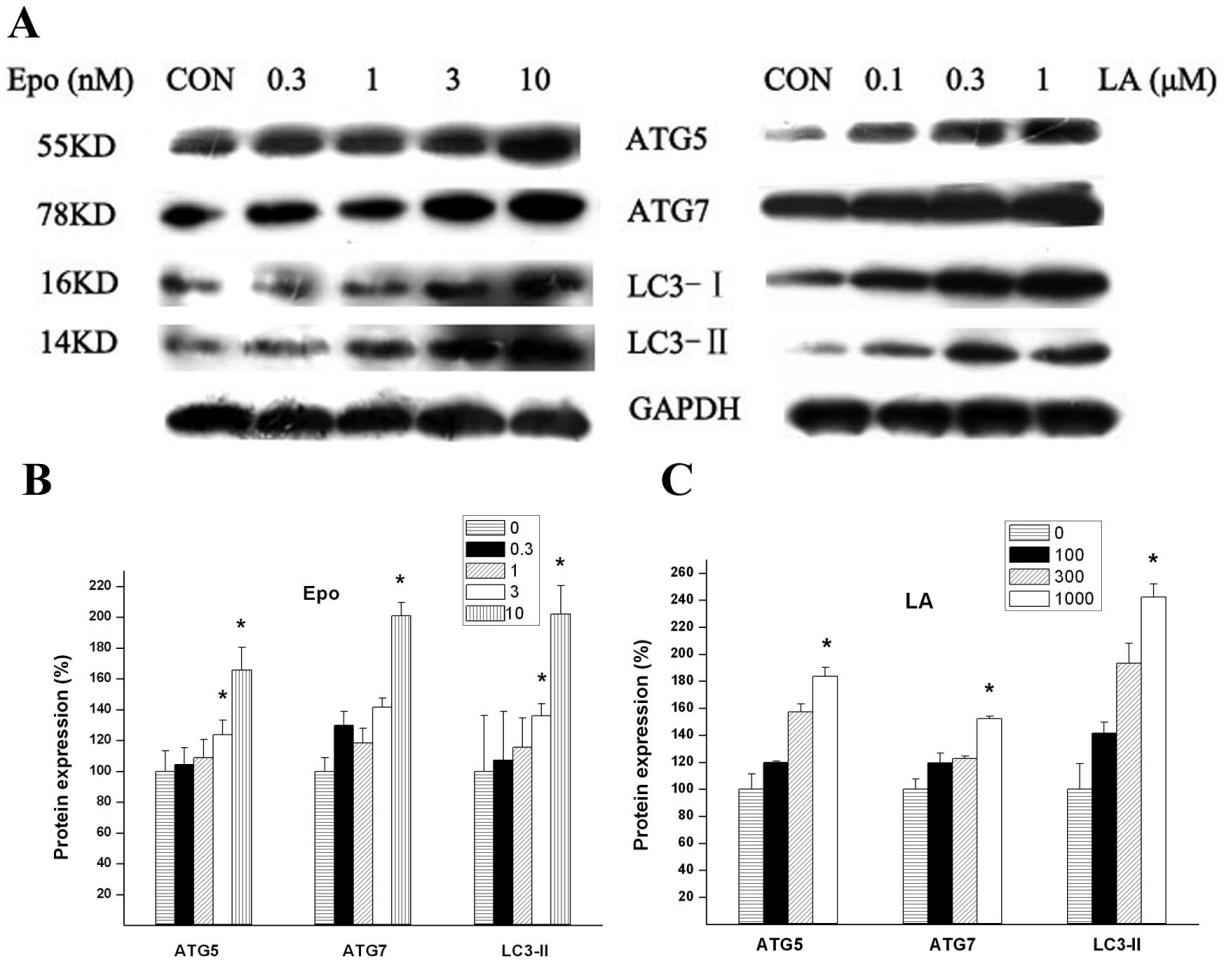
LA or Epo increased protein levels of ATG5, ATG7, and the conversion of LC3-I to LC3-II. **A**, ARPE-19 cells were treated with Epo (0.3∼10 nM, left panel) or LA (100-1000 nM, right panel) for 18-24 h, and proteins were harvested and subjected to immunoblotting for ATG5, ATG7, LC3 and GAPDH. The blots shown were typical of at least triplicate experiments. The ratios of ATG5/GAPDH, ATG7/GAPHH, and LC3-II/GAPDH for Epo (**B**) or LA treatment (**C**) were mean (+ SEM) of at least triplicate experiments. The ratios for control were set as 100% and the values from treatment conditions were normalized to the control values. P<0.05 *vs* control.

**Figure 2:**
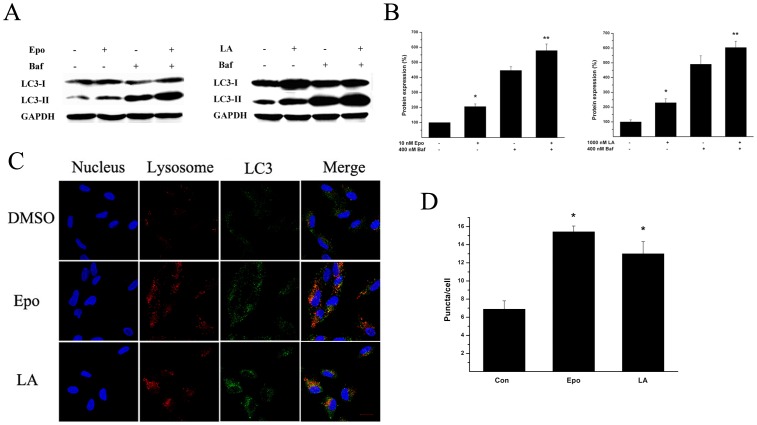
LA or Epo induced autophagic flux. **A**, ARPE-19 cells were treated with DMSO, Epo (10 nM), or LA (1 µM) for 18 h and Baf (400 nM) was added to the cultures for the final 4 h. Proteins were harvested and subjected to immunoblotting against LC3. The blots shown are typical of at least triplicate experiments. The optic densities were averaged and quantified in **B**, the values in control were set as 100% and the values in treated conditions were normalized to the control values. * P<0.05 *vs* control; **P<0.05 indicated that Epo or LA plus Baf differed significantly from Epo or LA treatments. **C**, ARPE-19 cells were treated with DMSO, Epo (10 nM), or LA (1 µM) for 18 h, and labeled with fluorescence as described in Methods, and imaged by confocal laser microscope. The images shown were typical of the images from five non-contiguous fields in each dish from triplicate experiments. Scale bar, 20 µM. The LC3-positive puncta overlaying labeled lysosome for Epo, or LA treatment, and sham condition were averaged from 20 cells and quantified in **D**. * P<0.05 *vs* control. Blue, DAPI-labeled nuclei; Red, lyso tracker-labeled lysosome; Green, FITC-labeled LC3. The merged images were shown in the most right column and the orange-stained cells indicated LC3, co-localized with lysosome.

To determine whether LA or Epo activate the autophagy pathway in RPE, we first examined the levels of Atg5 and Atg7 proteins, essential for autophagosome maturation, and measured the conversion of LC3 from LC3-I to LC3-II before and after LA or Epo treatment. 18–24 h treatment with LA (100∼1000 nM) or Epo (0.3∼10 nM) increased the protein levels of Atg5/Atg7, and the conversion of LC3 (Fig. 1). To determine whether overproduction of Atg-related proteins by LA or Epo treatment was due to increased autophagosome formation or due to decreased autophagosome fusion with lysosome, saturated concentration of Baf, i.e. completely blocked autophagosome fusion with lysosome, was added to LA or Epo treated cultures at the final 4 h; this operation further increased the protein level of LC3-II (Figs. 2A, and 2B). This method to monitor autophagic flux was described previously [21]. To further confirm autophagic flux induced by LA or Epo treatment, we analyzed the co-localization of LC3 staining with lysosome. As shown in Fig. 2C, LA or Epo treatment increased LC3-positive puncta (3^rd^ column of the 2^nd^ and 3^rd^ rows) compared to the sham treatment (3^rd^ column of the 1^st^ row) and LA or Epo treatment further increased the co-localization between LC3-positive puncta and labeled lysosome (4^th^ column of the 2^nd^ and 3^rd^ row) compared to the sham treatment (4^th^ column of the 1^st^ row). Together, the results suggest that increased protein levels of Atg-related proteins by LA or Epo treatment are not due to blockage of autophagic flux, but due to increased formation of autophagosome.

### 2. Inhibition of PI3K/Akt/mTOR pathway by LA or Epo

**Figure 3:**
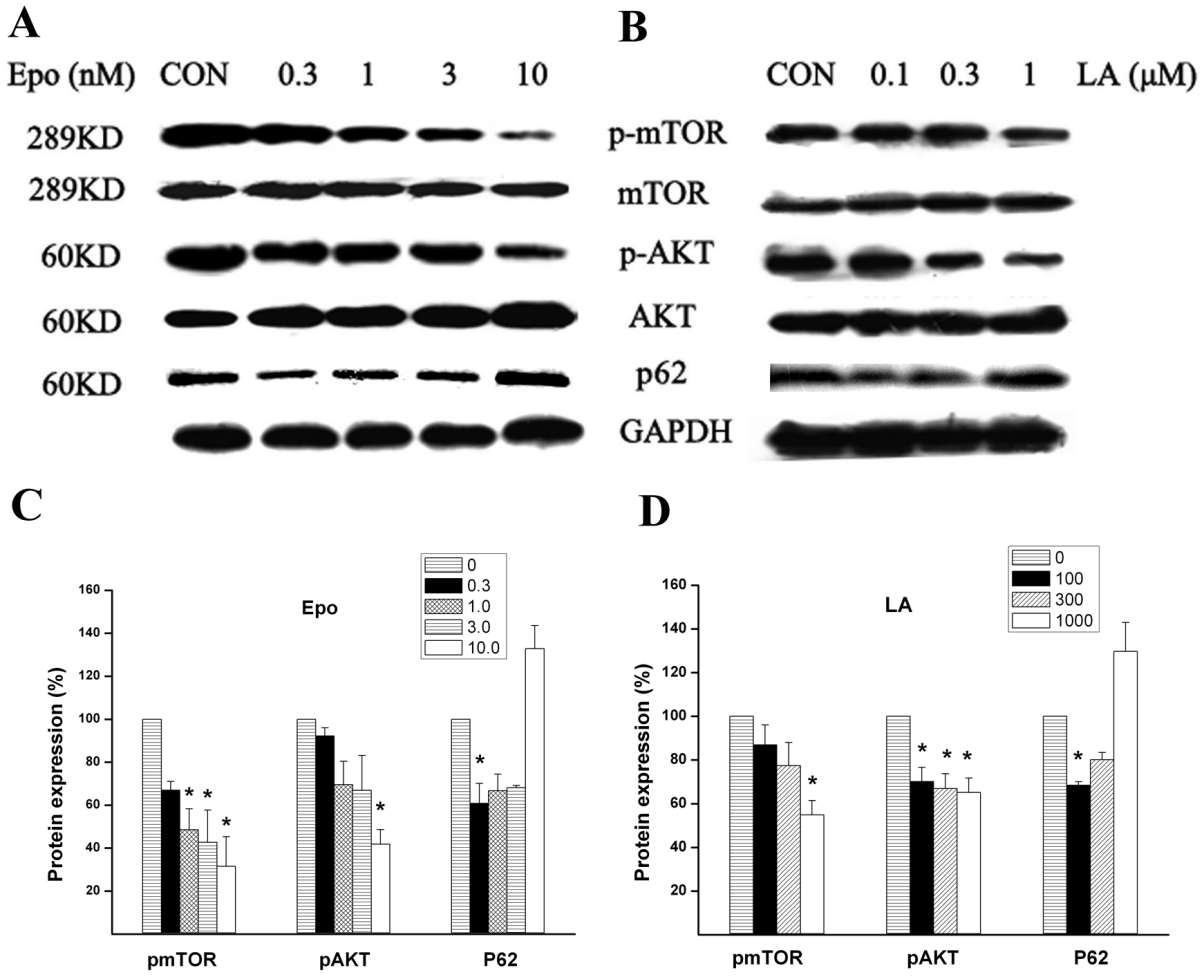
LA or Epo decreased phospho-AKT and phospho-mTOR protein levels. ARPE-19 cells were treated with Epo (0.3∼10 nM) (**A**) or LA (100–1000 nM) (**B**) for 18–24 h, and proteins were harvested and subjected to immunoblotting for mTOR, phospho-mTOR(p-mTOR), AKT, phospho-AKT(p-AKT), p62. The optical density ratios (mTOR/GAPDH, AKT/GAPDH, p-mTOR/GAPDH, p-AKT/GAPDH, p62/GAPDH) for Epo (**C**) or LA treatment (**D**) were averaged from at least triplicate experiments and the ratios for the mTOR/GAPDH, AKT/GAPDH were not shown. The values for control were set as 100%; the values for treatment condition were normalized to the control values. *P <0.05 *vs* control.

Previous studies indicate that the PI3K/Akt/mTOR axis plays important roles in autophagy inhibition, especially in starvation-induced autophagy; inhibition of mTOR is one way to activate autophagy [22], [23]. Therefore, we tested whether LA or Epo affect the PI3K/Akt/mTOR pathway. Both LA (especially at 1 µM) and Epo (especially at 10 nM) reduced levels of phospho-AKT and phospho-mTOR but had little effect on the levels of AKT and mTOR (Figs. 3A and 3B). Since p62 protein, also named as sequestosome1(SQSTM1), is degraded by autophagy, it may be used as a marker for autophagic flux [24]. To confirm that LA or Epo induce autophagy, we tested p62 level in LA or Epo-treated cultures and the changes of p62 indicated a biphasic pattern, i.e. p62 was reduced at low doses but gradually increased with raised concentrations of LA or Epo (Figs. 3A and 3B). The p62 level induced by LA or Epo,especially at relatively high concentrations, is probably the mixture of autophagy degradation and proteasome inhibition, i.e. autophagy degradation reduces p62 but proteasome inhibition increases p62. Several studies indicated p62 overexpression occurs under the conditions of proteasome inhibition [25], [26].

### 3. Bafilomycin A1(Baf) reversed the protective effects of LA or Epo against oxidative injuries in ARPE-19 cells

**Figure 4:**
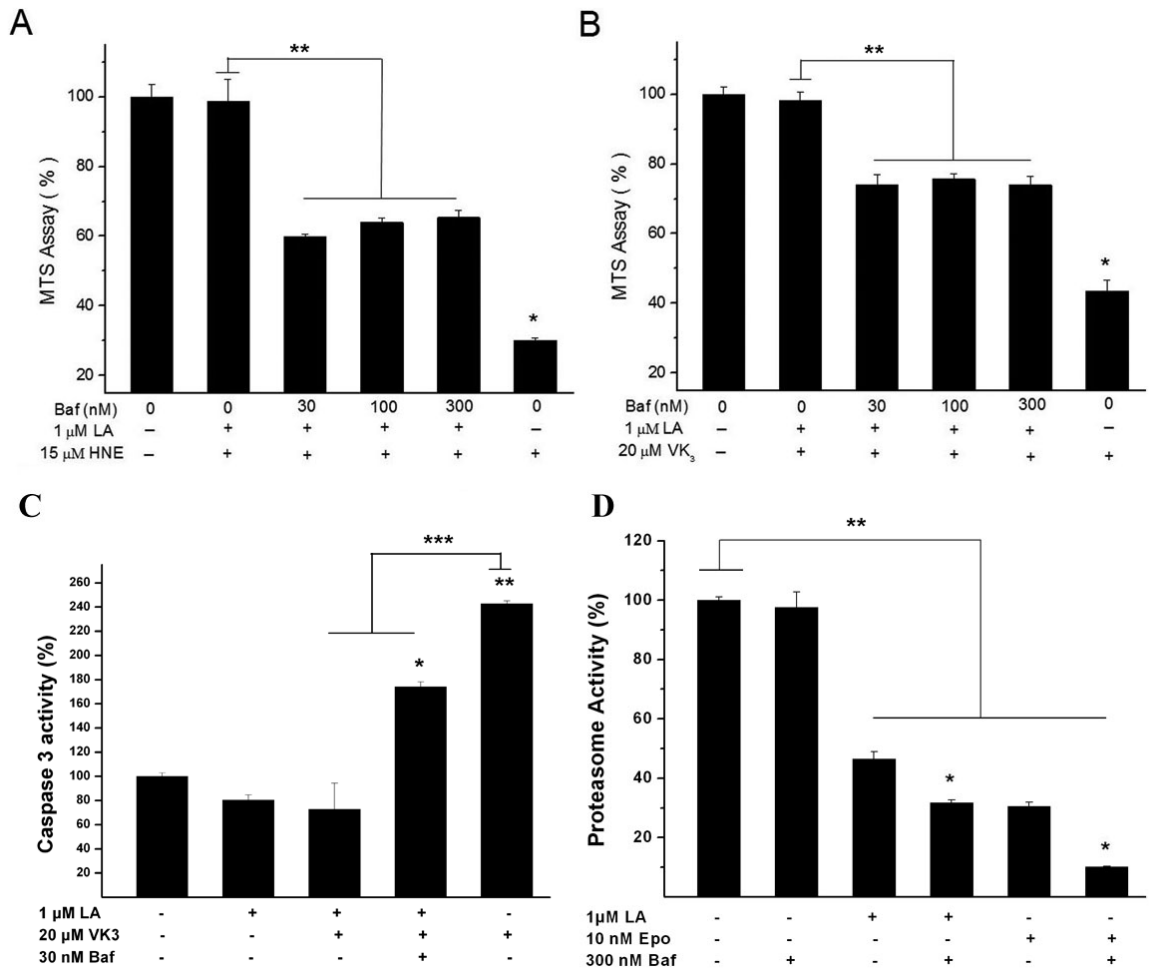
Bafilomycin A1 (Baf) reversed the protective effect of LA against HNE or VK3 in ARPE-19 cells. Cultures were pre-treated with LA and the indicated concentrations of Baf (30∼300 nM) for 18 h before 18 h exposure to HNE (15 µM) (**A**) or VK3 (20 µM) (**B**). MTS assay was used to measure cell viability (**A**, **B**) and caspase-3 activity assay to measure apoptosis (**C**) at the end of the 18 h HNE or VK3 treatment. In MTS assay, *P<0.05 *vs*. control, ** P<0.05 indicated that the three combinatorial treatment including HNE or VK3, LA, and Baf differed significantly from cultures treated by HNE plus LA or VK3 plus LA; in caspase-3 assay, ** P<0.05 *vs*. control, ***P, *P <0.05 indicated that the three combinatorial treatment including VK3, LA, and Baf differed significantly from either VK3 or VK3 plus LA treatment respectively. **D**, ARPE-19 cells were treated by Baf (300 nM), LA (1 µM), Epo (10 nM), LA plus Baf, or Epo plus Baf for 18 h, and then subjected to chymotrypsin-like proteasome activity assay as described in Methods. *P<0.05 indicated significant difference between LA and LA plus Baf or between Epo and Epo plus Baf treatment; **P<0.05 indicated significant difference between control and treatment conditions except by Baf. All the values in control cultures (**A**, **B**,**C**,**D**) were set at 100% and the values in treated cultures were normalized to the control values. All the results shown are mean (± SEM) of at least triplicate experiments in quadruplicate cultures.

**Figure 5:**
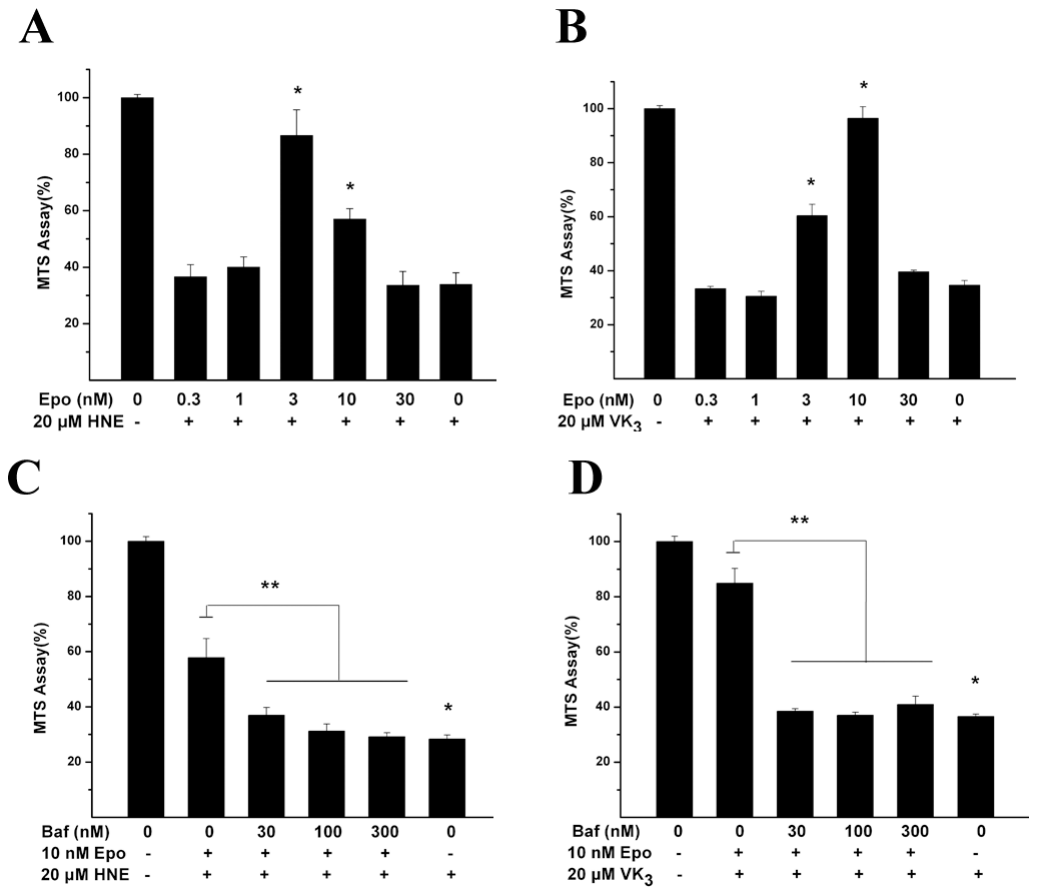
Bafilomycin A1 (Baf) reversed the protection of Epo against HNE or VK3. Cultures were pre-treated with the indicated concentrations of Epo for 18 h before 18 h exposure to HNE (15 µM) (**A**) or VK3 (20 µM) (**B**); or the cultures were pre-treated with Epo (10 nM) and the indicated concentrations of Baf (30∼300 nM) for 18 h before 18 h exposure to HNE (15 µM) (**C**) or VK3 (20 µM) (**D**). MTS assay was used to measure cell viability at the end of the 18 h HNE or VK3 treatment. The results shown in A, B, C, and D are mean (± SEM) of at least three independent experiments in quadruplicate cultures. The values in the control cultures were set at 100% and the survivals in treated cultures were normalized to the control values. * P<0.05 vs. control, ** P<0.05 indicated that the three combinatorial treatment including HNE or VK3, Epo, Baf differed from either Epo plus VK3 or Epo plus HNE treatment.

We have demonstrated that LA protects against oxidative injuries in ARPE-19 cells [18]. However, the detailed mechanism remains unclear. Recent studies indicated that proteasome inhibition could activate autophagy [7]. Therefore, we examined whether Baf, an inhibitor of vacuolar-type H^+^-ATPase [27] that suppresses autophagic flux, could attenuate the protective effects of LA. First, we confirmed that Baf alone did not alter the viability of RPE cells. RPE cells remained viable after 24 h treatment with Baf (3∼300 nM) (Fig. S1). We next treated cultures with 1 µM LA, a paradigm that results in the maximal reduction in oxidative injury [18], As expected, 18 h pretreatment with LA (1 µM) completely blocked the toxicity of HNE or VK3 (Figs. 4A, and 4B), which is consistent with our previous study [18]. At the tested concentrations, LA inhibited proteasome activity as reported in our previous study [18]; co-addition of Baf with LA or Epo showed additive effects on proteasome inhibition (Fig. 4D), which are compatible with previous study [11], although Baf alone, at the maximal dose applied, 300 nM, did not change proteasome activity. Co-application of Baf (30∼300 nM) for 18 h with LA partially reversed the beneficial effects of LA (Figs. 4A, and 4B). To confirm the consistency between the results for MTS and apoptosis assays, we examined caspase-3 activity in the above cultures. VK3 treatment significantly increased caspase-3 activity compared to sham cultures, whereas co-addition of LA with VK3 reduced caspase-3 activity to the basal level; the values of capspase3 activity by the three combinatorial treatment including Baf, LA, and VK3 were in the middle of the values for VK3 and LA plus VK3 treatments (Fig. 4C). In summary, the results for apoptosis assay were compatible with the MTS results, thus, only MTS assay was used to examine the survival status in the rest study. To confirm that the protective effect by LA is a general phenomenon for irreversible proteasome inhibitors, we tested another irreversible proteasome inhibitor, Epo. A 22 h pretreatment with Epo (0.3∼10 nM) significantly blocked the toxicity of HNE, with a maximal protective effect at a concentration of 3 nM Epo against HNE-induced injury and 10 nM against VK3-induced injury (Figs. 5A, and 5B). At these concentrations, Epo inhibited proteasome activity significantly (Fig. S4). Co-application of Baf (30∼300 nM) with the Epo during the 18 h pretreatment period completely reversed the protective effects of Epo against HNE or VK3-induced cell death (Figs. 5C, and 5D).

### 4. Knockdown of Atg7 attenuated the protective effects of LA or Epo

**Figure 6:**
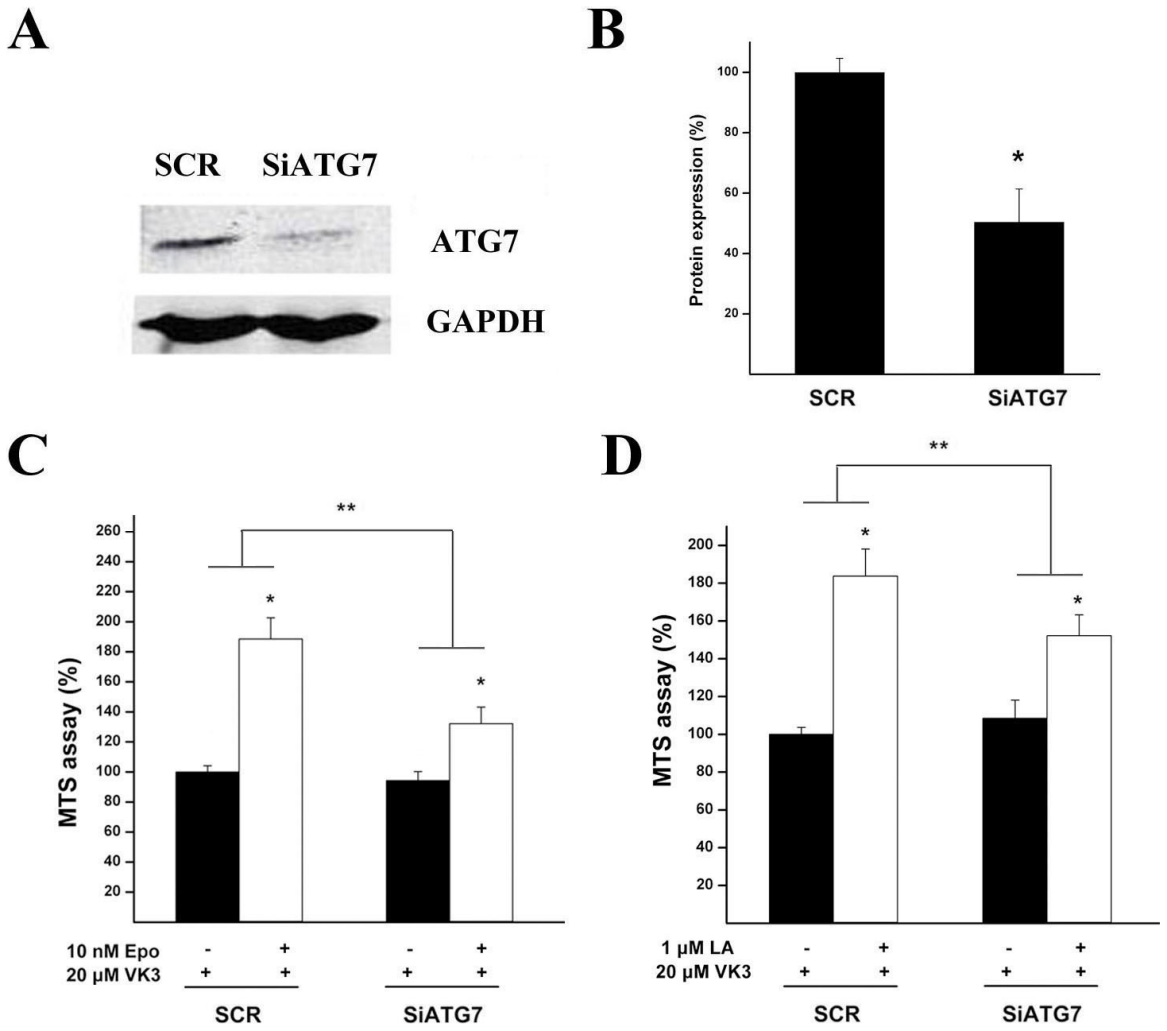
Knockdown of Atg7 attenuated the protective effect of LA or Epo. ARPE-19 cells were transfected with scramble siRNA (SCR), or Atg7-specific siRNA (SiATG7), continued to be cultured for 24 h, followed by pre-treatments with Epo (10 nM, **C**) or LA (1 µM, **D**), or sham treatment for 18–24 h, and then subjected to VK3 (20 µM) treatment for 18 h. After the 18 h VK3 treatment, the cultures were subjected to western blot analyses (**A**) or MTS assay (**C**, **D**). The knockdown effects by siRNA were quantified in **B**, *P<0.05 indicated significant difference between the knockdown effect of SiATG7 and SCR. In **C**, **D**, *P<0.05 indicated significant differences between LA or Epo treatment and LA or Epo plus VK3 treatments; ** P<0.05 indicated significant differences between the protective effects of LA or Epo treatment in SCR group and those in SiATG7 group. All the results were averaged from at least triplicate experiments and the values in control were set as 100% and the values in treated conditions were normalized to the control values.

Considering the possible non-specific effects of LA or Epo treatment, we examined the effects of LA or Epo on the toxicity of VK3 in Atg7-knockdown ARPE-19 cells. Transfection with Atg7-specific siRNA (SiATG7) reduced Atg7 in ARPE-19 cell cultures beyond 50% of the level in the cultures transfected with scramble siRNA (SCR) (Figs. 6 A, and 6B). Knockdown of Atg7 significantly reduced, but not completely blocked the protective effect of LA or Epo compared to the cultures transfected with scramble siRNA (Figs. 6C, and 6D). Thus, the protection by LA or Epo against VK3 toxicity also involves autophagy-independent mechanism in addition to up-regulating autophagy.

## Discussion

Our results demonstrate that the proteasome inhibitors LA or Epo activated the autophagy pathway, as measured by increased level of autophagosome proteins ATG5 and ATG7, increased conversion of LC3-I to LC3-II, and increased autophagic flux. We also demonstrated that LA or Epo inhibited the PI3K/Akt/mTOR pathways, which is a possible way how LA or Epo induce autophagy. We further demonstrated that autophagy inhibitor, Baf, completely reversed the protective effects of low doses of proteasome inhibitor Epo (Figs. 5A, and 5B), as well as for the effects of MG-132 (Fig. S2); by contrast, Baf partially reversed the cytoprotective effects of LA (Figs. 4A, and 4B). Therefore, the cytoprotective effect of Epo may be mediated exclusively through activation of autophagy, while other mechanisms may contribute to the effects of LA. For example, we have found that LA can ameliorate the reduction of glutathione levels seen after oxidative injury in ARPE-19 cells (unpublished data); this effect would be unlikely to be affected by Baf. We further demonstrated that the protective effects of LA or Epo were significantly attenuated under the condition of knockdown of ATG7. Overall, our results suggest that LA or Epo reduced vulnerability to oxidative injuries at least in part by activation of the autophagy, possibly through inhibition of PI3K/Akt/mTOR signaling. Considering the non-specific effects from the relatively high doses of LA or Epo, e.g. interfering with autophagy substrate degradation (Figs. 3A, and 3B) or reducing endoplasmic reticulum quality-control system [28], low doses of LA or Epo, even autophagy enhancers, rapamycin and its analogs [29], would be better candidates to be used against oxidative injury in RPE cells.

Previous studies have suggested that peroxisome proliferator-activated receptor alpha (PPARα) antagonist partially reversed the protective effect of low doses of MG-132 against oxidative injuries [18]. Thus, PPAR family antagonists were also tested on the effects of Epo against HNE or VK3. PPARα antagonist GW6471, but not PPARγ antagonist GW9662, reversed the protective effects of Epo in a dose-dependent manner; at 20 µM, the effects of GW6471 reached the maximal (Fig. S3). In summary, low doses of MG-132, Epo or LA protected RPE from oxidative injury via activating autophagy and PPAR pathway activation also contributes to the anti-oxidative roles for MG-132 or Epo, but not for LA. The reasons that LA could not activate PPAR pathway in ARPE-19 cells are currently unclear and is under investigation. Some studies indicated that PPAR activation could induce autophagy [30], [31], which may explain that both PPARα antagonist and Baf could reverse the protective effects of MG-132 or Epo against oxidative injuries, summarized from our previous and current studies.

The cytoprotective effects of Epo against HNE and VK3 toxicity disappeared at high Epo concentrations (Figs. 5A, and 5B); this is similar to observations using proteasome inhibitors LA and MG-132 in our previous study [18]. The loss of cytoprotection at higher concentrations of these inhibitors may reflect inherent toxicity of high concentrations of these inhibitors. The optimal concentration of Epo against HNE was 3 nM in contrast with 10 nM against VK3 (Figs. 5A, and 5B). The difference probably results from the differential inherent toxicity for HNE and VK3, e.g. HNE is conjugated to proteins and/or induces oxidative stress [32] in contrast with VK3, majorly as an oxidative stressor. It is reasonable to think that Epo, as a potent and selective proteasome inhibitor, may induce higher extent of protein aggregation at 10 nM than at 3 nM, thus caused more toxic effect with HNE at relatively higher concentration.

Several recent studies have demonstrated interactions between the proteasome and autophagy degradative pathways. For example, increased expression of histone deacetylase (HDAC6) reduces degeneration in flies with genetic inhibition of the UPS and in a fly model of spinobulbar muscular atrophy; the effect of HDAC6 is mediated by an increase in autophagy [33]. We did not detect a change of the levels of HDAC6 in LA/Epo treated ARPE-19 cells (data not shown). There are other mechanisms for cross-talk between the UPS and autophagy pathways. For example, proteasome inhibitions induce accumulation of misfolded proteins which activates the unfolded protein response pathway; this pathway works via inositol-requiring enzyme 1 (IRE1), an ER transmembrane protein kinase/endoribonuclease, to activate a number of pathways, including autophagy. This is a JNK-dependent pathway in several cell types [8], [14]. We demonstrated here that PI3K/Akt/mTOR pathway was inhibited by LA and Epo; inhibition of mTOR contributes to autophagy activation in some situations [23]. Therefore, we inferred that inhibition of mTOR pathway by LA or Epo, may contribute to their induction of autophagy.

Phagocytosis and degradation of shed outer segments by the RPE cells are critical for survival of photoreceptors - - this process involves degradation of shedded discs by autophagy and lysosomal degradation [34], [35], [36]. Atrophy or even death of retinal pigment cells (RPEs) and photoreceptors [37], [38] are the major pathological changes in dry age-related macular degeneration (AMD). Oxidative stress may play a role in RPE dysfunction in AMD [39]. The results of this study suggest that interactions between the UPS and autophagy might be a potential therapeutic target in AMD and other disorders where oxidative stress may play a role.

## Supporting Information

Figure S1 **Baf did not compromise human RPE survival.** ARPE-19 cultures were treated with indicated concentrations of Baf (3∼300 nM) for 24 h. MTS assay was used to measure cell viability at the end of treatment. The values in the sham-washed control cultures were set at 100% and the survivals in treated cultures were normalized to the control values. The results shown are mean (± SEM) of at least triplicate experiments in quadruplicate cultures.
(TIF)

Figure S2 **Baf reversed the protections of MG-132 against HNE.** Cultures were pre-treated with MG-132 (30 nM) and the indicated concentrations of Baf (30∼300 nM) for 18 h before 18 h exposure to HNE (15 µM). MTS assay was used to measure cell viability at the end of the 18 h HNE treatment. The values in control cultures were set at 100% and the survivals in treated cultures were normalized to the control values. The results shown are mean (± SEM) of at least three independent experiments in quadruplicate cultures. *P<0.05 *vs*. control, ** P<0.05 indicated that the three combinatorial treatment including 4-HNE, MG-132, and Baf (100, 300 nM) differed significantly from cultures treated by 4-HNE plus MG-132.
(TIF)

Figure S3 **PPARα antagonist GW6471, but not PPARγ antagonist GW9662, reversed the protection of Epo against VK3.** Cultures were pre-treated with Epo (10 nM) and the indicated concentrations of GW6471 (10∼20 µM) (**A**) or GW9662 (1∼30 µM) (**B**) for 18 h before 18 h exposure to VK3 (20 µM). MTS assay was used to measure cell viability at the end of the 18 h VK3 treatment. The values in control cultures were set at 100% and the survivals in treated cultures were normalized to the control values. The results shown are mean (± SEM) of at least three independent experiments in quadruplicate cultures. *P<0.05 *vs*. control, ** P<0.05 indicated that the three combinatorial treatment including VK3, Epo, and GW6471 differed significantly from cultures treated by VK3 plus GW6471.
(TIF)

Figure S4 **Epo inhibited proteasome activity in a dose-dependent manner.** ARPE-19 cell cultures were treated with different concentrations of Epo (0.3∼30 nM) for 18 h, the cultures were harvested and chymotrypsin-like proteasome activity was measured. The results were averaged from at least triplicate cultures, and the values from treated cultures were normalized to those in the control cultures (proteasome activity 100%). * P<0.05 *vs.* control.
(TIF)
