# Supplementary figures and images for: Proteasome Inhibitors Activate Autophagy Involving Inhibition of PI3K-Akt-mTOR Pathway as an Anti-Oxidation Defense in Human RPE Cells
Source: PLoS One. 2014 Jul 25;9(7):e103364. doi: 10.1371/journal.pone.0103364 (PMC4111584; doi:10.1371/journal.pone.0103364)

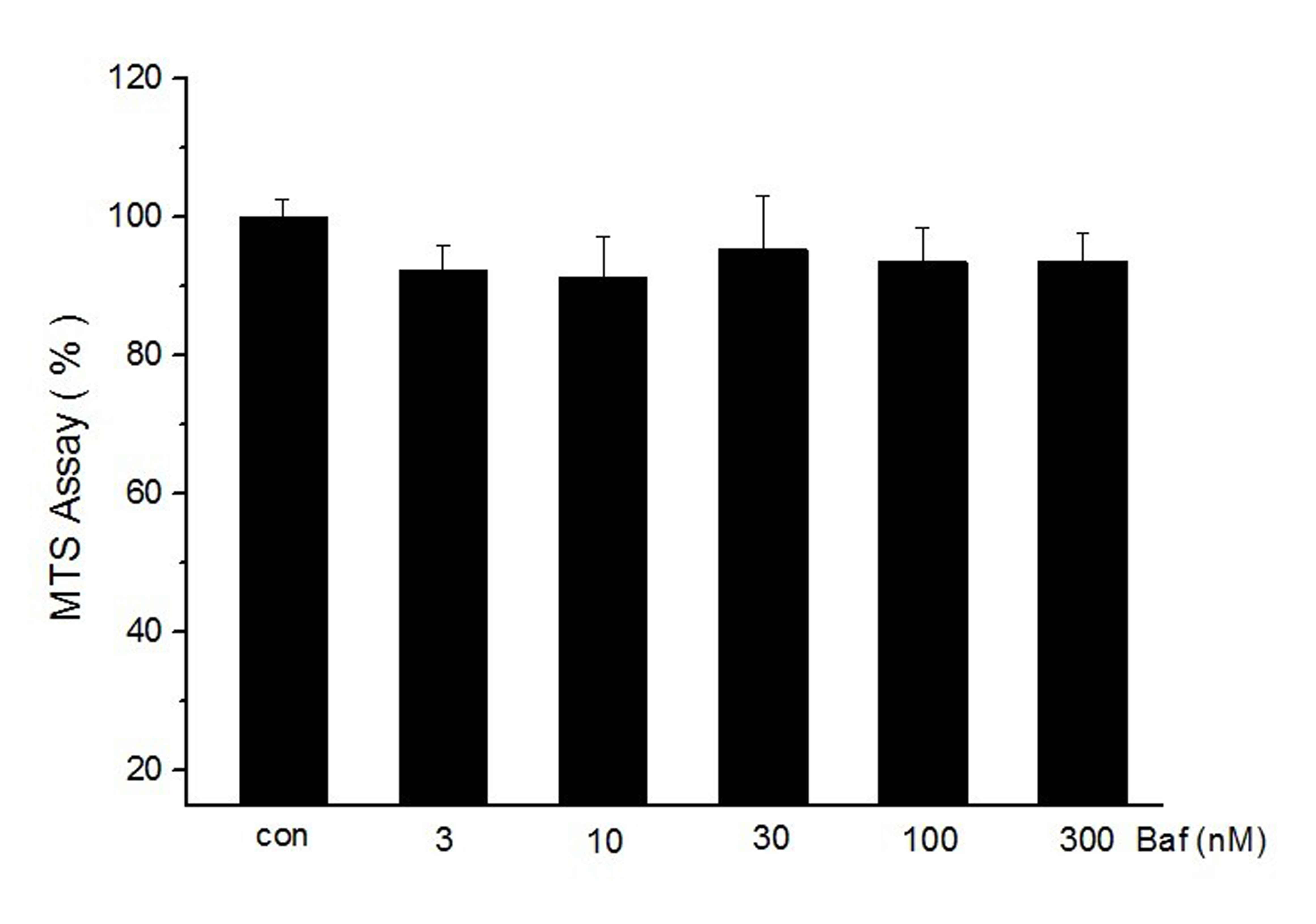

Supplement: Figure S1 — Baf did not compromise human RPE survival. ARPE-19 cultures were treated with indicated concentrations of Baf (3∼300 nM) for 24 h. MTS assay was used to measure cell viability at the end of treatment. The values in the sham-washed control cultures were set at 100% and the survivals in treated cultures were normalized to the control values. The results shown are mean (± SEM) of at least triplicate experiments in quadruplicate cultures. (TIF) [file pone.0103364.s001.tif]

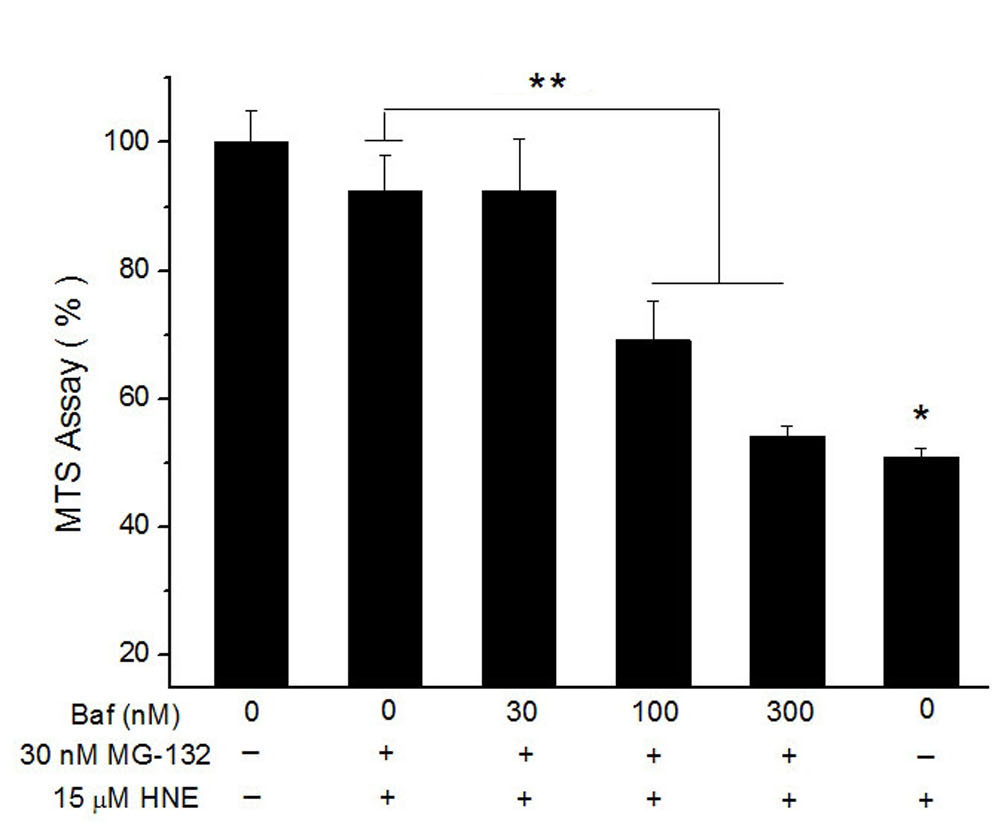

Supplement: Figure S2 — Baf reversed the protections of MG-132 against HNE. Cultures were pre-treated with MG-132 (30 nM) and the indicated concentrations of Baf (30∼300 nM) for 18 h before 18 h exposure to HNE (15 µM). MTS assay was used to measure cell viability at the end of the 18 h HNE treatment. The values in control cultures were set at 100% and the survivals in treated cultures were normalized to the control values. The results shown are mean (± SEM) of at least three independent experiments in quadruplicate cultures. *P<0.05 vs. control, ** P<0.05 indicated that the three combinatorial treatment including 4-HNE, MG-132, and Baf (100, 300 nM) differed significantly from cultures treated by 4-HNE plus MG-132. (TIF) [file pone.0103364.s002.tif]

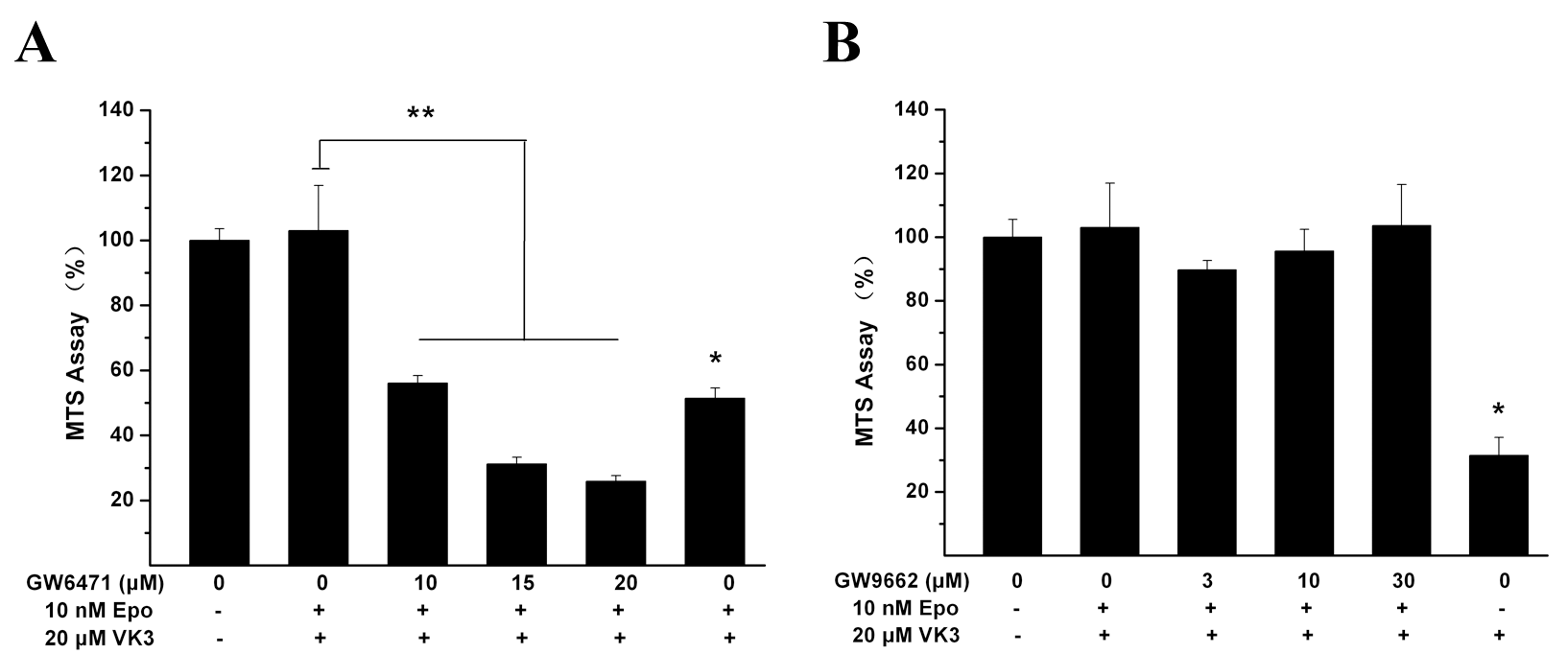

Supplement: Figure S3 — PPARα antagonist GW6471, but not PPARγ antagonist GW9662, reversed the protection of Epo against VK3. Cultures were pre-treated with Epo (10 nM) and the indicated concentrations of GW6471 (10∼20 µM) (A) or GW9662 (1∼30 µM) (B) for 18 h before 18 h exposure to VK3 (20 µM). MTS assay was used to measure cell viability at the end of the 18 h VK3 treatment. The values in control cultures were set at 100% and the survivals in treated cultures were normalized to the control values. The results shown are mean (± SEM) of at least three independent experiments in quadruplicate cultures. *P<0.05 vs. control, ** P<0.05 indicated that the three combinatorial treatment including VK3, Epo, and GW6471 differed significantly from cultures treated by VK3 plus GW6471. (TIF) [file pone.0103364.s003.tif]

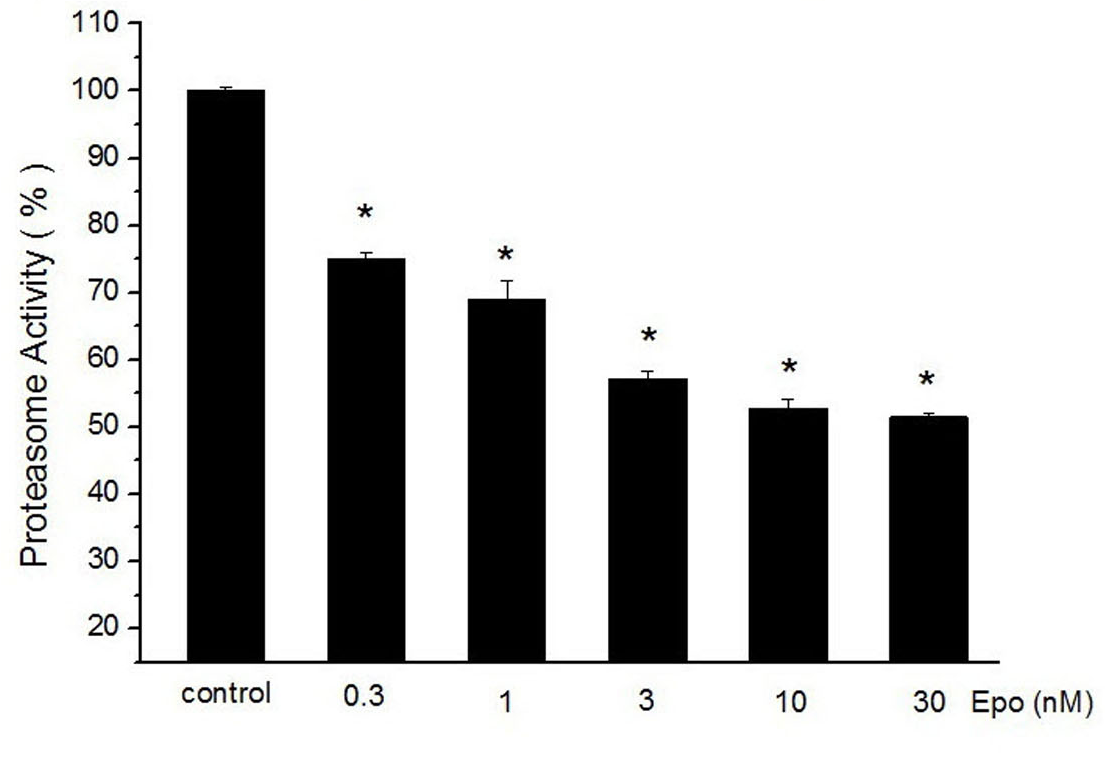

Supplement: Figure S4 — Epo inhibited proteasome activity in a dose-dependent manner. ARPE-19 cell cultures were treated with different concentrations of Epo (0.3∼30 nM) for 18 h, the cultures were harvested and chymotrypsin-like proteasome activity was measured. The results were averaged from at least triplicate cultures, and the values from treated cultures were normalized to those in the control cultures (proteasome activity 100%). * P<0.05 vs. control. (TIF) [file pone.0103364.s004.tif]
